# Autoantibodies as diagnostic markers and potential drivers of inflammation in ulcerative colitis

**DOI:** 10.1371/journal.pone.0228615

**Published:** 2020-02-12

**Authors:** Henrika Jodeleit, Lisa Milchram, Regina Soldo, Gabriel Beikircher, Silvia Schönthaler, Omar Al-amodi, Eckhard Wolf, Florian Beigel, Andreas Weinhäusel, Matthias Siebeck, Roswitha Gropp

**Affiliations:** 1 Department of General, Visceral und Transplantation Surgery, Hospital of the Ludwig-Maximilian-University Munich, Munich, Germany; 2 Austrian Institute of Technology GmbH (AIT), Giefinggasse, Wien, Austria; 3 Institute of Molecular Animal Breeding and Biotechnology, Laboratory for Functional Genome Analysis (*LAFUGA*), Gene Center, LMU Munich, Munich, Germany; 4 Department of Medicine II, Hospital of the Ludwig-Maximilian University Munich, München, Germany; Universita degli Studi di Napoli Federico II, ITALY

## Abstract

To date, no comprehensive analysis of autoantibodies in sera of patients with ulcerative colitis has been conducted. To analyze the spectrum of autoantibodies and to elucidate their role serum-IgG from UC patients (n = 49) and non-UC donors (n = 23) were screened by using a human protein microarray. Screening yielded a remarkable number of 697 differentially-reactive at the nominal 0·01 significance level (FDR<0·1) of the univariate test between the UC and the non-UC group. CD99 emerged as a biomarker to discriminate between both groups (p = 1e-04, AUC = 0·8). In addition, cytokines, chemokines and growth factors were analyzed by Olink’s Proseek® Multiplex Inflammation-I 96×96 immuno-qPCR assay and 31 genes were significant at the nominal 0.05 level of the univariate test to discriminate between UC and non-UC donors. MCP-3, HGF and CXCL-9 were identified as the most significant markers to discriminate between UC patients with clinically active and inactive disease. Levels of CXCL10 (cor = 0.3; p = 0.02), CCL25 (cor = 0.25; p = 0.04) and CCL28 (cor = 0.3; p = 0.02) correlated positively with levels of anti CD99. To assess whether autoantibodies are detectable prior to diagnosis with UC, sera from nine donors at two different time points (T-early, median 21 months and T-late, median 6 months) were analyzed. 1201 features were identified with higher reactivity in samples at time points closer to clinical UC presentation. *In vitro*, additional challenge of peripheral mononuclear cells with CD99 did not activate CD4+ T cells but induced the secretion of IL-10 (-CD99: 20.21±20.25; +CD99: 130.20±89.55; mean ±sd; p = 0.015). To examine the effect of CD99 *in vivo*, inflammation and autoantibody levels were examined in NOD/ScidIL2Rγ^null^ mice reconstituted with PBMC from UC donors (NSG-UC). Additional challenge with CD99 aggravated disease symptoms and pathological phenotype as indicated by the elevated clinical score (-CD99: 1·85 ± 1·94; +CD99: 4·25 ± 1·48) and histological score (-CD99: 2·16 ± 0·83; +CD99: 3·15 ± 1·16, p = 0·01). Furthermore, levels of anti-CD99 antibodies increased (Control: 398 ± 323; mean MFI ± sd; Ethanol + PBS: 358 ±316; Ethanol + CD99: 1363 ± 1336; Control versus Ethanol + CD99: p = 0.03). In a highly inflammatory environment, frequencies of pro-inflammatory M1 monocytes (CD14+ CD64+: unchallenged 8.09±4.72; challenged 14.2±8.62; p = 0.07; CD14+ CD1a+: unchallenged 16.29 ±6.97; challenged 43.81±14.4, p = 0.0003) increased and levels of autoantibodies in serum decreased in the NSG-UC mouse model. These results suggest that autoantibodies are potent biomarkers to discriminate between UC and non-UC and indicate risk to develop UC. In an inflammatory environment, auto-antibodies may promote the pathological phenotype by activating M1 monocytes in the NSG-UC animal model and also in patients with UC.

## Introduction

Ulcerative colitis (UC) belongs to the chronic inflammatory diseases with unknown etiology. It is presently thought that a combination of genetic, environmental and microbial factors is responsible for an uncontrolled immune response [1]. For a long time this immune response was considered as characterized and driven by the typical Th2 cytokine interleukin (IL)-13 (for review see [2]). At present, it is disputed whether a Th1 or Th2 immune response dominates inflammation in UC as aberrant expression of circulating Th1, T17 and cytotoxic (c) T cells has been demonstrated in patients with UC [3]. Furthermore, polymorphism in the IL-10 locus confers risk for developing UC suggesting that regulation of intestinal tolerance is disturbed in UC patients [4, 5]. We recently took on a different view considering the inflammatory process in UC as an uncontrolled wound healing process [6]. This hypothesis assumes that epithelial damage induces the release of signals to evoke Th1, Th2 and Th17 characterized inflammatory responses that ensure the protection of the mucosa and ultimately results in repair of the colon.

In order to gain a better understanding of the inflammatory processes an animal model was developed which is based on NOD-scid IL2Rγ^null^ (NSG) mice reconstituted with PBMC from diseased UC-individuals (NSG-UC model) [7, 8]. Challenge of mice with ethanol evoked symptoms and phenotype similar to the human disease and treatment with infliximab promoted a remodeling condition characterized by increased fibrosis. As in the human disease, subtypes of monocytes are significant markers of inflammation, especially the FcγR1 receptor bearing pro-inflammatory monocytes (CD14+ CD64+) [8, 9]. Of note, the development of severe symptoms could only be observed if PBMC from seriously affected patients were used for reconstitution, suggesting that immune cells from UC patients bear a memory lacking in non-diseased individuals. This observation led to the hypothesis that a breach of tolerance might be one cause for the development of symptoms and phenotype. To date, autoantibodies directed against perinuclear neutrophilic proteins, neutrophil cytoplasmatic proteinase PR-3, epithelial cells of the mucosa (antiGP-2, GAB), tropomyosin isoform 5 and pancreatic cells [10–14] have been detected in patients with colitis. However, no comprehensive analysis has been conducted to examine the expression of autoantibodies in UC patients and their potential role in inflammation. In this study we examine the expression of autoantibodies and markers of inflammation in UC patients as compared to non-diseased individuals and correlate expression levels with the immunological profile of patients. As CD99 emerged as a significant autoantigen, CD99 was selected to examine the role of that autoantigen *in vitro* and *in vivo*. Data presented here show that autoantibodies are significant markers of UC and that autoimmunity might promote inflammation in the NSG-UC model.

## Material and methods

### Detection of autoantibodies

Written consent was given by all donors and the study was approved by the Institutional Review Board (IRB) of the Medical Faculty at the University of Munich (120–15).

Protein-microarray analysis was conducted using AIT’s 16k protein-microarray presenting proteins recombinantly expressed in E.coli from human 15286 cDNA expression clones. These so called UNIPEX clones represent full length cDNA clones, which were selected from expression clones derived from human fetal brain (N = 7383) as well as T-cells, Lung- and Colon- tissue (N = 7903; personal information provided by RZPD). These UNIPEX clones represent 6369 different human proteins (each protein presented by 2 or more different clones), for which 5449 have been annotated with a gene-symbol, when the cDNA sequence perfectly matched to database entries. Antibody profiling using purified IgG from sera was conducted as described previously [15–17].

In short: IgG was purified from sera (UC patients: n = 49; non-UC controls: n = 23) using MelonGel and standardised concentrations of 0·2 mg IgG/mL applying 450μL per microarray were used for antibody-profiling. Patient’s IgG bound onto antigens presented on microarrays were detected by a fluorescently labelled anti-human detection antibody. Background–corrected median relative fluorescent intensities (RFI) from microarray images were log2 transformed. The median normalized Log2-RFI values were then bio-statistically evaluated with BRB Array Tools Version 4.5.0 (National Cancer Institute, USA; http://linus.nci.nih.gov/BRB-ArrayTools.html).

Mouse antibody experiments were performed with a Luminex protein bead array representing 192 recombinantly expressed proteins produced in E. coli cDNA clones, deduced from 16k experiments. IgG was purified from mouse sera with the MelonGel Spin Plate Kit according manufacturer’s instructions. 50 μL of concentration adjusted IgG (0·05 mg/mL in PBS 1%BSA) were used for each well and processed as previously described [18]. Blank corrected median fluorescence intensities (MFI) were log2 transformed and subjected to bioinformatic analysis.

### Detection of cytokines, chemokines and growth factors

For detection of cytokines, chemokines and growth factors serum samples (49 UC patients, 15 non-UC controls) were analysed using Olink’s Proseek® Multiplex Inflammation-I96×96 immunoassay (Olink Biosciences Uppsala, Sweden). The experiment was performed according to the manufacturer’s instructions. In brief, samples were incubated over night at 4°C with two PEA probes, followed by a preamplification step and the detection using Fluidigm’s 96.96 Dynamic Array IFC (Fluidigm, CA, USA). The raw data was prepared with respect to Olink’s guidelines using the Proseek Universal DataPreprocessing file Version 1 and the Inflammation I protein list (both files can be downloaded from Olink’s webpage; http://www.olink.com/data-you-can-trust/data-generation-qc/#macro). The normalized protein expression units expressed in terms of log2- values were then bio-statistically evaluated with BRB Array Tools Version 4.4.1 (National Cancer Institute, USA).

### Isolation of PBMC and engraftment

60 mL of peripheral blood were collected from the arm vein of patients suffering from UC in trisodium citrate solution (S-Monovette, Sarstedt, Nürnberg, Germany). The blood was diluted with Hank’s balanced salt solution (HBSS, Sigma Aldrich, Deisenhofen, Germany) in a 1:2 ratio and 30 mL of the suspension were loaded onto LeucoSep tubes (Greiner Bio One, Frickenhausen, Germany). PBMC were separated by centrifugation at 400 g for 30 minutes and no acceleration. The interphase was extracted and diluted with phosphate buffered saline (PBS) to a final volume of 40 mL. Cells were counted and centrifuged at 1400 g for 5 minutes. The cell pellet was resuspended in PBS at a concentration of 4 x 10^6^ cells in 100μL.

Six to eight week old NOD.cg-Prkdc^SCID^ Il2rg^tm1Wjl^/Szj mice (abbreviated as NSG) were engrafted with 100 μL cell suspension into the tail vein on day 1.

### Animal study protocol

Animal studies were approved by the committees of the government of Upper Bavaria, Sachgebiet 54 –Tierschutz, Germany (55.2-2-1-54-2532-74-15) and performed in compliance with German Animal Welfare Laws.

NOD IL-2Rγ^null^ mice were obtained from Charles River Laboratories (Sulzfeld, Germany). Mice were kept under specific pathogen free conditions in individually ventilated cages in a facility controlled according to the Federation of Laboratory Animal Science Association (FELASA) guidelines. Following engraftment (day 1) mice were presensitized by rectal application of 150 μL of 10% ethanol on day 8 using a 1mm cat catheter (Henry Schein, Hamburg, Germany). The catheter was lubricated with XylocainGel 2% (AstraZeneca, Wedel). Post application mice were kept at an angle of 30° to avoid ethanol dripping. On day 15 and 18 mice were challenged by rectal application of 50% ethanol following the protocol of day 8. All rectal applications were performed under general anaesthesia using 4% isofluran. Mice were sacrificed under kentamin and xylacin anaesthesia by cervical dislocation on day 21. Number of animals used: Control n = 8, ethanol n = 10. To study the effects of antigens mice were engrafted and presensitized following the protocol described above. On days 15, 20 and 25 mice were challenged with 150 μL of 30% ethanol. CD99 (10 μg in 200 μL PBS, Creative Biomart, Shirley, USA) were applied intraperitoneally on day 15, 20 and 25. PBS was applied to the challenged control group. Mice were sacrificed on day 28. No of animals used: Control n = 8, ethanol n = 8, ethanol + CD99 n = 8.

Post challenge with ethanol, animals were monitored daily. Scores used to assess the wellbeing of the animals included weight loss, body composure, behaviour, injuries, appearance of the fur.

### Clinical activity score

In UC patients, clinical activity was defined by the Simple Clinical Colitis Activity Index [19].

In the mouse model, assessment of colitis-severity was performed daily according to the following scoring system: Loss of body weight: 0% (0), 0–5% (1), 5–10% (2), 10–15% (3), 15–20% (4). Stool consistency: formed pellet (0), loose stool or unformed pellet (2), liquid stools (4). Behaviour: normal (0), reduced activity (1), apathy (4) and ruffled fur (1). Body posture: Intermediately hunched posture (1), permanently hunched posture (2). The scores were added daily into a total score with a maximum of 12 points per day. Animals who suffered from weight loss > 20%, rectal bleeding, rectal prolapse, self-isolation or a severity score > 7 were euthanized immediately and not taken into count. All scores were added for statistical analysis.

### Isolation of human leukocytes

To isolate human leucocytes from murine spleen, spleens were minced and cells filtrated through a 70 μL cell strainer (Greiner Bio-One, Frickenhausen) followed by centrifugation at 1400 g for 5 minutes and resuspended in FACS buffer (1 x PBS, 2mM EDTA, 2%FCS). For further purification cell suspensions were filtrated using a 35 μm cell strainer (Greiner Bio-One, Frickenhausen) and then labelled for flow cytometry analysis.

For isolation of lamina propria mononuclear cells (LPMC) a protocol modified of Weigmann et al., 2007 was used. The washed and minced colon was pre-digested in an orbital shaker with slow rotation (40g) at 37° Celsius for 1 x 20 minutes in predigestion solution containing 1x HBSS (Thermo Scientific, Darmstadt, Deutschland), 5mM EDTA, 5% FCS, 100 U/mL Pencillin-Streptomycin (Sigma-Aldrich Co.,St. Louise USA). Epithelial cells were removed by filtering through a nylon filter. After washing with RPMI the remaining colon pieces were digested for 2 x 20 minutes in digestion solution containing 1 x RPMI (Thermo Scientific, Darmstadt, Deutschland), 10% FCS, 1 mg/mL Collagenase A (Sigma-Aldrich Co.,St. Louis USA), 10 KU/mL Dnase I (Sigma-Aldrich Co.,St. Louise USA), 100 U/mL Pencillin-Streptomycin (Sigma-Aldrich Co.,St. Louis USA) in an orbital shaker with slow rotation (40g) at 37° Celsius [20].

Isolated LPMC were centrifuged at 1400 g for 5 minutes and resuspended in FACS buffer. Cell suspensions were filtrated one more time using a 35 μm cell strainer (Greiner Bio-One, Frickenhausen) Germany for further purification before labelling the cells for flow cytometry analysis.

### Flow cytometry analysis

Labelling of human leucocytes was performed according to S6 Table.

All antibodies were purchased from Biolegend (San Diego, USA) and used according to the manufacturer’s instructions. Flow cytometry was performed using a BD FACS Canto II^™^ and analysed with FlowJo 10·1-Software (FlowJo LLC, Oregon, USA).

### Histological analysis

Samples from distal parts of the colon were fixed in 4% Formaldehyde for 24 hours, before storage in 70% ethanol and were routinely embedded in paraffin. Samples were cut into 3 μm sections and stained with haematoxylin and eosin (H&E). Epithelial erosions were scored as follows: no lesions (0), focal lesions (1), multifocal lesions (2), major damage with involvement of basal membrane (4). Inflammation was scored as follows: infiltration of few inflammatory cells into the Lamina propria (1), major infiltration of inflammatory cells into the Lamina propria (2), confluent infiltration of inflammatory cells into the Lamina propria (3), infiltration of inflammatory cells including tunica muscularis (4). Fibrosis was scored as follows: focal fibrosis (1), multifocal fibrosis and crypt atrophy (2). The presence of edema, hyperemia and crypt abscess was scored with 1 additional point in each case. The scores for each criterion were added into a total score ranging from 0 to 13. Images were taken with a Zeiss AxioVert 40 CFL camera. Figures show representative longitudinal sections in original magnification. In Adobe Photoshop CS6 a tonal correction was used in order to enhance contrasts within the pictures.

### Cell culture

PBMC of healthy individuals and UC patients were isolated. The cell pellet was resuspended in RPMI (Thermo Fisher Scientific, Waltham, MA, USA) at a concentration of 1 x 10^6^ cells / mL. Additionally 500 μL RPMI with 10% FCS and 1% Penicillin-Streptomycin (Sigma-Aldrich, St. Louis, MO, USA) were added to each well and sample. To compare T cell activation 10 μg / mL CD99 (Creative Biomart, Shirley, USA) was added according to the protocol by Zaunders et al ([21]. Wells containing PBMC and RPMI or PBMC, RPMI anti CD3 (1μg /mL) and anti CD28 antibodies (5μg/mL; Biolegend, (San Diego, USA) served as negative and positive controls, respectively. Cells were incubated for 48 hours. The content of each well was centrifuged at 1400 g for 5 minutes. The pellet was resuspended in FACS buffer and labelled for flow cytometry.

### Detection of amino acids

Samples were prepared according to the manufacturer’s instruction. Following incubation of 100 μL of serum with internal standards for 5 min, 25 μL of 15% 5-sulfosalicylic acid was added and samples were centrifuged at 9000g for 15 min at 4°C. Supernatants were filtered through a 0.2 μm membrane and 75 μL of lithium loading buffer was added. Samples were analyzed by the amino acid analyzer Biochrom 30+ (Biochrom Ltd, Cambridge, UK).

### Statistics

Statistical analysis was performed with R: A language and environment for statistical computing. (R Foundation for Statistical Computing, Vienna, Austria. URL https://www.R-project.org/; RRID:SCR_001905) and BRB Array Tools (https://brb.nci.nih.gov/BRB-ArrayTools/). Variables were represented with mean, standard deviation, median, and IQR values. A two-sided Student’s t-test and a confidence level = .95 was used to compare binary groups and for more than two groups ANOVA followed by TukeyHSD was conducted.

PCA models were validated by ordinal regression.

## Results

### Autoantibody expression and immunology profiles in UC patients

To pursue a comprehensive analysis of autoantibodies, blood samples were collected from 49 UC patients and 23 non-affected individuals. For basic demographics see Table 1.

**Table 1:** Baseline demographics, duration of disease and therapy of patients.

	**UC** **N = 49**	**Active UC** **N = 19**	**Non-UC** **N = 23**
**Age (years)**			
** Mean (SD)**	38·5 (15·6)	36.13	36·7 (15·9)
** Range**	24–74	19–71	21–59
**Gender (% male)**	46	42	42
**Duration of UC (years)**			
** Mean (SD)**	11·6 (9·53)	11.46 (10.97)	
** Range**	1–40	1–39	
**SCCAI**			
** Mean (SD)**	3·04 (2·79)	6.42 (2.19)	
** Range**	0–13	5–13	
**Treatment (current)**			
** TNFα-blocker**	20	6	
** Glucocorticoids**	13	8	
** Mesalazine**	26	9	
** Immuno-suppressive**	6	3	
** No**	10	3	
**Atopic Dermatitis**			3

**Fig 1:**
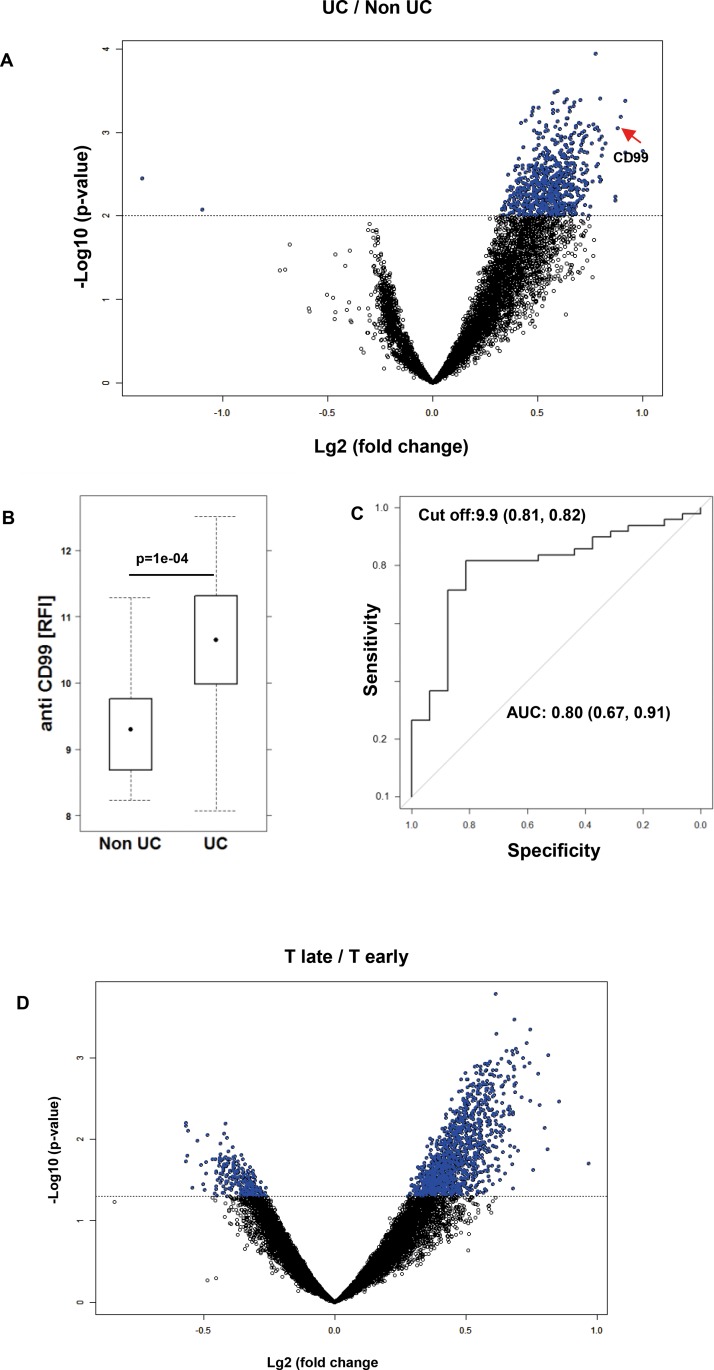
Autoantibodies as biomarkers to discriminate between UC and non-UC donors. (A) Increased autoantibody reactivity in UC patients displayed as a volcano plot. Purified serum IgG of UC (49) and non-UC (23) patients were subjected to protein-microarray analysis of UC vs non-UC. Antigenic reactivity in UC versus non-UC is presented. Significantly differentially reactive antigens are given in blue (n = 697; p<0.01; x-axis: “-log10 p value”; y-axis shows the log2 –fold change between classes-). **(**B) Expression levels of anti-CD99 antibodies in UC (n = 49) and non-UC (n = 23) donors depicted as a boxplot diagram. RFI, Log2 of median relative fluorescent intensity. Boxes represent upper and lower quartiles and whiskers represent variability. For comparison of non-UC versus UC, a Student’s t-test was performed. (C) ROC curve for anti-CD99 antibodies in differentiating UC and Non-UC patients. Sample sizes: non-UC n = 19, UC n = 49. AUC: Area under the curve; Cut of value indicates the optimal ratios of sensitivity and specificity. (D) Increased autoantibody reactivity in donors prior to diagnosis with UC presented as volcano plot. IgG (purified from plasma) from 9 different donors at two different time points (T-late: median 6 months prior to diagnosis; T-early: median 13 months prior to T-late) were examined. Antigenic reactivity in T-early versus T-late is presented. Significantly differentially reactive antigens are given in blue (n = 1201; p<0.01; x-axis: “-log10 p value”; y-axis shows the log2 –fold change between classes.

Sera were prepared as described in Materials and Methods. The clinical activity score was assessed by using the simple clinical colitis activity index (SCCAI) [19]. Immunglobulin G purified from sera of UC and non-UC donors were subjected to high-density protein microarray antibody profiling using AIT’s 16k protein-microarray [15]. Much to our surprise, analysis of autoantibody expression revealed 697 autoantigens that were significantly different at the nominal 0·01 significance level (FDR<0·1) of the univariate test between the UC group and the group of non-affected individuals (Fig 1A, the complete list of autoantibodies is shown in S1 Table). CD99 could be identified as a biological marker with high potential to discriminate between UC and non-UC subjects (AUC = 0·8) (Fig 1B and 1C). At a level of 9.9 RFI 81% of UC patients were diagnosed positively, whereas 82% of non-UC patients were tested negative. No changes of autoantibody levels was observed when samples were tested for IgA using the same 16k protein-array.

To examine whether autoantibodies can be detected prior to diagnosis of UC sera from blood donors from the Bavarian Red Cross—biobank were examined. From the entire biobanked samples 9 donors were identified who repeatedly donated blood and who were excluded from donation at some time point due to diagnosis with UC. Prediagnostic samples from 9 different donors were analyzed and were separated into two groups: Samples taken within 4–11 months (T-late, median = 6 months before clinical diagnosis) and samples taken within 9–24 months prior to diagnosis (T-early, median 13 months to sample T-late). Samples were matched with 33 healthy controls from the same biobank matched for age, gender and time points. Samples were subjected to the same analysis as described before. When applying class comparison in a paired manner (single feature significance level of p<0·05), 1452 features of 14938 features (remaining antigens on microarrays upon quality filtering–excluding spots derived from non-variant antigens, and controls) were significantly reactive and 1201 (of 1452) showed increased reactivity in the timepoint “T-late” vs “T-early” in paired analysis of prediagnostic UC-patients as shown in the volcano plot (Fig 1D, the complete list of autoantibodies is shown in S2 Table). Fold change increase of antibody levels in samples taken at time points closer to diagnosis indicate a higher antigenic reactivity in 1201 out of 1452 significant features. Comparing the 1452 differentially reactive antigens (IgG isolated from plasma) with the 697 autoantigens identified in the “UC (diseased) versus Control sample study” (IgG from serum) we find an overlap of n = 244 antigens (Odds ratio 4.28; 95% CI: 3.59 to 5.11; chi2 p-value < 2.2e-16). Subsetting the data to the 1452 features found significant in the “prediagnostic T3 vs Tearly UC”-plasma sample study, class comparison defines an overlap of n = 227 antigens, and 213 thereof are again found with significant higher reactivity in these prediagnostic UC vs control samples (Odds ratio 1.8562; 95% CI: 1.6013 to 2.1516; chi2 p-value = 1.307e-12; Class comparison results). Thus from 2 independent sample types–serum and plasma and in 2 independent experimental studies conducted with >1y time difference, and using different sources/biobanks of samples, we could confirm significant overlaps of differentially reactive antigenic proteins, thus confirming findings that antibody profiles are changed drastically in UC during disease onset and remain changes during disease. From these results we concluded that inflammation in UC is accompanied by a general leakiness in tolerance.

**Fig 2:**
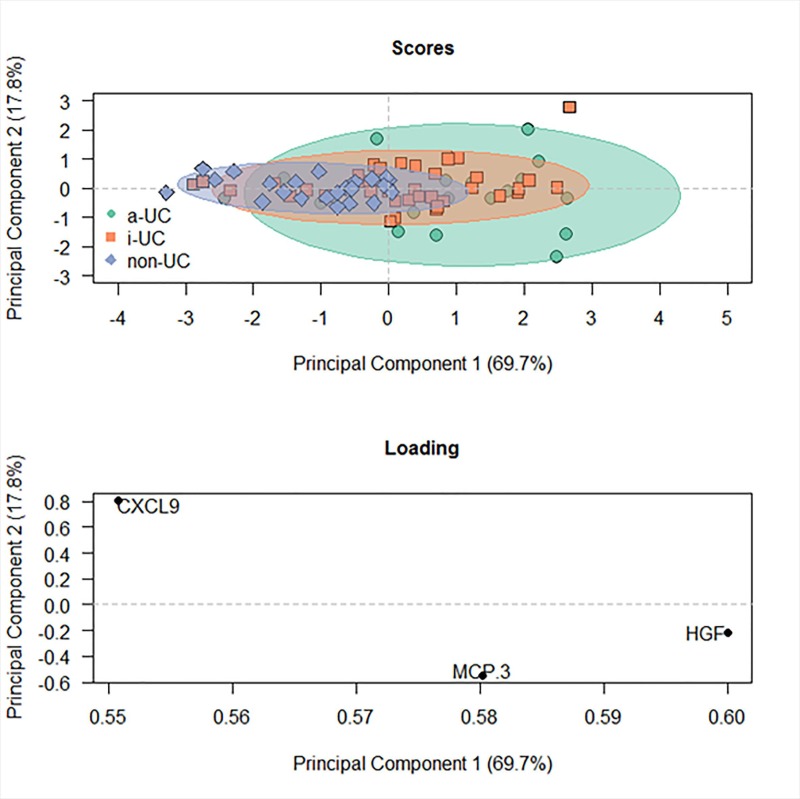
MCP-3, HGF and CXCL9 as markers to discriminate between non-UC, inactive UC and active UC presented as Principal Component Analysis. Serum samples were subjected to Olink’s Proseek® Multiplex Inflammation-I96×96 immunoassay. Sample size: non-UC n = 23; inactive UC (SCCAI<5) n = 36, active UC (SCCAI>5) n = 19. Significance was evaluated by using ordinal regression analysis.

In order to examine whether the expression level of autoantibody against CD99 was correlated with cytokine-, chemokine or growth factor levels a comprehensive analysis was performed using Olink’s Proseek® Multiplex Inflammation-I 96×96 immuno-qPCR assay. 31 genes were significant at the nominal 0.05 level of the univariate test (Table 2), most of which are common inflammatory markers. In addition to pro-inflammatory cytokines such as IL-8, IL-17A, IL-7, IL12B, IL-18, OSM, and IL-6, chemokines were identified which are responsible for attracting T helper cells (Monocyte chemoattractant protein (MCP) -3, MCP-4), activated T cells (chemokine CXC motif ligand (CXCL) 9, CXCL10, CXCL11), naïve T cells (CC chemokine ligand (CCL) 19), monocyte/ macrophages (macrophage inflammatory protein (MIP) -1α, CXCL10, CCL23, MCP-4), eosinophils, basophils, neutrophils and NK cells (MCP-3, MCP-4, CXCL9, CCL11, CXCL10, CXCL11) and B cells (CXCL9, CXCL10, CXCL11, CCL19, MIP-1α), as well as IgA+ plasma cells (CCL25). Hepatic growth factor (HGF) has previously been identified by our group as an indicator of acute inflammation [22]. Elevated levels of vascular endothelial growth factor A (VEGFA) and fibroblast growth factor (FGF) are most probably indicators of the ongoing remodeling of the colon. Principal component analysis revealed that levels of MCP-3, CXCL9 and HGF alone are powerful markers to discriminate between non-UC individuals and UC patients with inactive or active disease (active disease was defined by a SCCAI score ≥ 5). As shown in Fig 2, the non-UC group clustered closely whereas the group with active disease were widely spread indicating a high heterogeneity in this group. The group of sera from inactive UC patients clusterd in the middle. The model was validated by ordinal regression (PrChi value = 4 e-0·6). This model discriminated significantly between active (SCCAI <5) and inactive disease (SCCAI ≥ 5, p = 0·003) and inactive disease and non-UC (p = 0·03).

**Table 2:** Serum marker analysis of UC patients versus non-UC donors. Sample sizes: UC = 49, non-UC = 23.

	Parametric p-value	FDR	Geom mean of intensities in UC patients	Geom mean of intensities in non-UC donors	Fold-change	Symbol
1	3·28e-05	0·00151	8·17	5·15	1·59	MCP-3
2	3·91e-05	0·00151	185·8	99·67	1·86	CXCL9
3	4·91e-05	0·00151	229·31	157·83	1·45	HGF
4	0·0001263	0·00259	6·93	4·44	1·56	MIP-1 alpha
5	0·000141	0·00259	11·38	7·86	1·45	CDCP1
6	0·0002304	0·00294	207·52	99·04	2.1	IL-8
7	0.0002658	0.00294	259.97	187.07	1·39	CCL11
8	0.0002755	0.00294	345.3	202.39	1·71	CXCL10
9	0.0002875	0.00294	775.23	574.1	1·35	CCL23
10	0.0003397	0.00313	348.8	181·79	1·92	CXCL11
11	0.0006626	0.00554	9.09	5.41	1·68	TGF-alpha
12	0.0008533	0.0062	3.22	2.32	1·39	IL-17A
13	0.0008766	0.0062	104.36	87.02	1.2	IL-10RB
14	0.0009597	0.00631	806.44	529.94	1.52	CCL19
15	0.0011439	0.00702	125.19	95.16	1.32	IL-18R1
16	0.0019023	0.0109	6.43	4.25	1.51	IL-6
17	0.0026739	0.0145	14.71	7.95	1.85	IL-10
18	0.0033233	0.017	43.61	27.49	1.59	OSM
19	0.0046164	0.0217	265.84	230.62	1.15	CSF-1
20	0.0047199	0.0217	4401.35	3194.31	1.38	VEGF-A
21	0.0074946	0.0328	7.21	5.8	1.24	SLAMF1
22	0.0093254	0.039	26.23	15.26	1.72	FGF-21
23	0.0137314	0.0549	123.6	91.36	1.35	MMP-10
24	0.0173102	0.0664	19.66	15	1.31	IL-7
25	0.0217397	0.0779	22.06	19.21	1.15	CD5
26	0.0220271	0.0779	144.33	162.13	0.89	DNER
27	0.0244441	0.0833	28.66	22.11	1.3	IL-12B
28	0.027095	0.089	1409.05	1145.89	1.23	OPG
29	0.0307398	0.0956	2030.75	1601.81	1.27	MCP-1
30	0.0311823	0.0956	206.45	175.6	1.18	IL-18
31	0.0368348	0.109	18.25	14.68	1.24	MCP-4

Correlation analysis of levels of anti CD99 antibody expression and level of inflammatory serum markers revealed that CXCL10, CCL25 and CCL28 were positively correlated with levels of anti CD99 antibodies as opposed to CCL23, oncostatin (OS) M and transforming growth factor (TGF)α, which were negatively correlated (Table 3). CCL23, OSM and TGFα, however; were positively correlated with the SCCAI score. It is noteworthy that CCL25 and CCL28 have been identified as mucosal chemokines and as attractants of intraepithelial lymphocytes, IgA+ plasma cells and mucosal memory cells.

**Table 3:** Correlation analyses of levels of inflammatory markers with levels of anti-CD99 antibodies (Anti CD99) and the SCCAI Score. Sample sizes: anti-CD n = 49. Numbers display Pearson’s product-moment correlation coefficients (cor-values), p-values, and 95% Confidence intervals (CI).

	Anti CD99			SCCAI		
Marker	Cor	p-value	95% CI	Cor	p-value	95% CI
CXCL10	0·3	0·02	0·061–1·0	-0·04		
CCL25	0·25	0·04	0·007–1·0	-0·17		
CCL28	0·3	0·02	0·07–1·0	0·09		
CCL23	-0·36	0·005	-1·0–0·13	0·32	0·01	0·095–1·0
OSM	-0·35	0·008	-1·0–0·11	0·45	0·0005	0·23–1·0
TGFα	-0·35	0·007	-1·0–0·12	0·18	0·09	

### The autoantigen CD99 elicits an anti-inflammatory response *in vitro*

**Fig 3:**
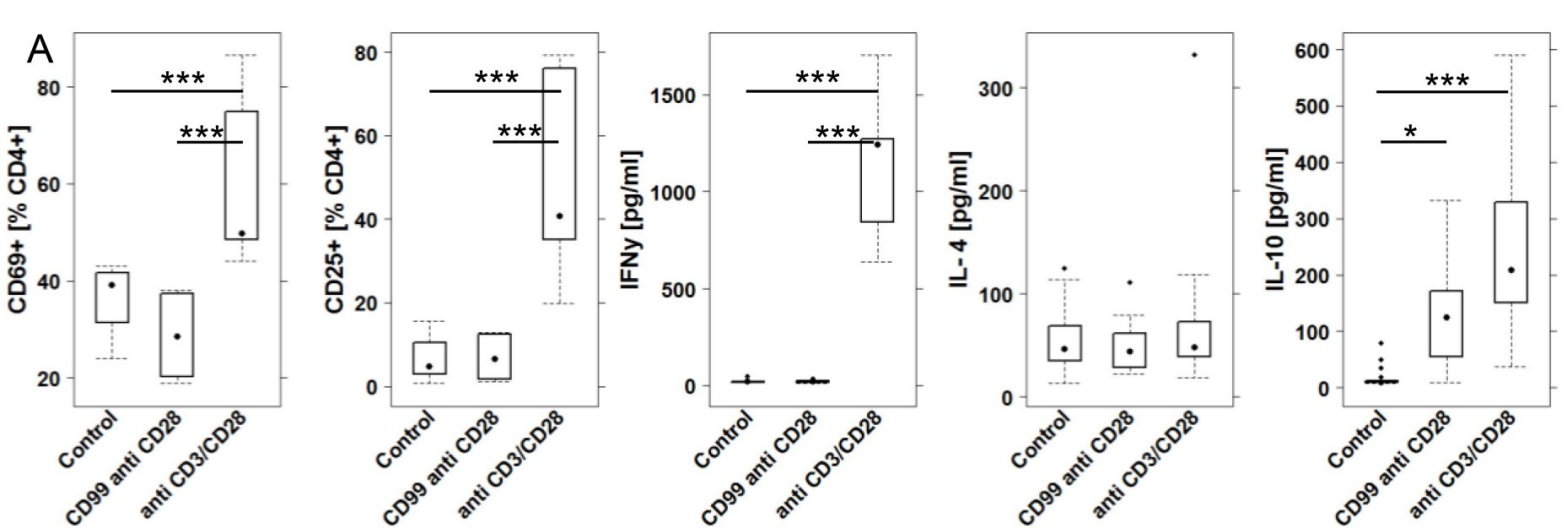
CD99 elicits an anti-inflammatory response *in vitro*. 1x10^6^ PBMC were incubated for 48h in 1.5 mL RPMI and subjected to flow cytometric analysis and supernatants to a multiplex Luminex assay. (A) Cells were incubated in the presence or absence of CD99 (10 μg / mL) and anti CD28 antibodies (5g /mL). Anti CD28 and anti CD3 antibodies (5 μg and 1μg/ mL, respectively) served as a positive control. Frequencies of activated CD4+ T cells and levels of IFNγ, IL-4 and IL-10 depicted as boxplot diagrams. Sample sizes: Donors n = 3 (Non-UC n = 1, UC n = 2). Control n = 6, CD99 n = 3. Boxes represent upper and lower quartiles, whiskers represent variability and outliers are plotted as individual points. For comparison of groups, ANOVA followed by Tukey’s HSD was conducted. Labels given on x-axes on the bottom row apply to all charts.

In order to gain a better understanding of the auto-reactivity, an *in vitro* analysis was performed. In UC patients, median anti-CD99 antibody levels were 1.84 fold increased (geometric mean of intensities, S1 Table). PBMC were isolated from three different donors: One non-UC donor and two UC patients. The UC patients had elevated levels of anti-CD99 antibodies (mean 11.4 and 10·7 [RFI], respectively). 1 x 10^6^ cells were incubated in RPMI medium in the absence or presence of anti CD3 (1μg / mL) and anti CD28 (5μg/mL) or CD99-protein (10 μg / mL) for 48h. Cells were isolated and subjected to flow cytometric analysis and cytokine expression analysis as described in Material and Methods (For definition of subtypes of leukocytes see S7 Table, for gating strategy see S1 Fig). To determine the activation of CD4+ cells we chose the early activation marker CD69 and CD25. Data revealed that the anti CD3 and anti CD28 antibodies evoked different responses in CD4+ T-cells as compared to CD99 treated cells (Fig 3, for complete data set see S3 Table). As expected, incubation with anti CD3 and anti CD28 antibodies induced the activation of CD4+ cells as shown by increased frequencies of CD4+ CD69+ and CD4+ CD25+ T-cells, whereas incubation with CD99 induced neither CD69+—nor CD25 expression in CD4+ T-cells. Cytokine expression analysis revealed that anti CD3 and anti CD28 antibodies evoked the secretion of IFNγ and IL-10, whereas CD99 addition to cell cultures induced solely the secretion of IL-10. No difference was observed between PBMC derived from Non-UC or UC donors.

### Additional challenge with CD99 aggravates disease symptoms and phenotype in the NSG-UC model

**Fig 4:**
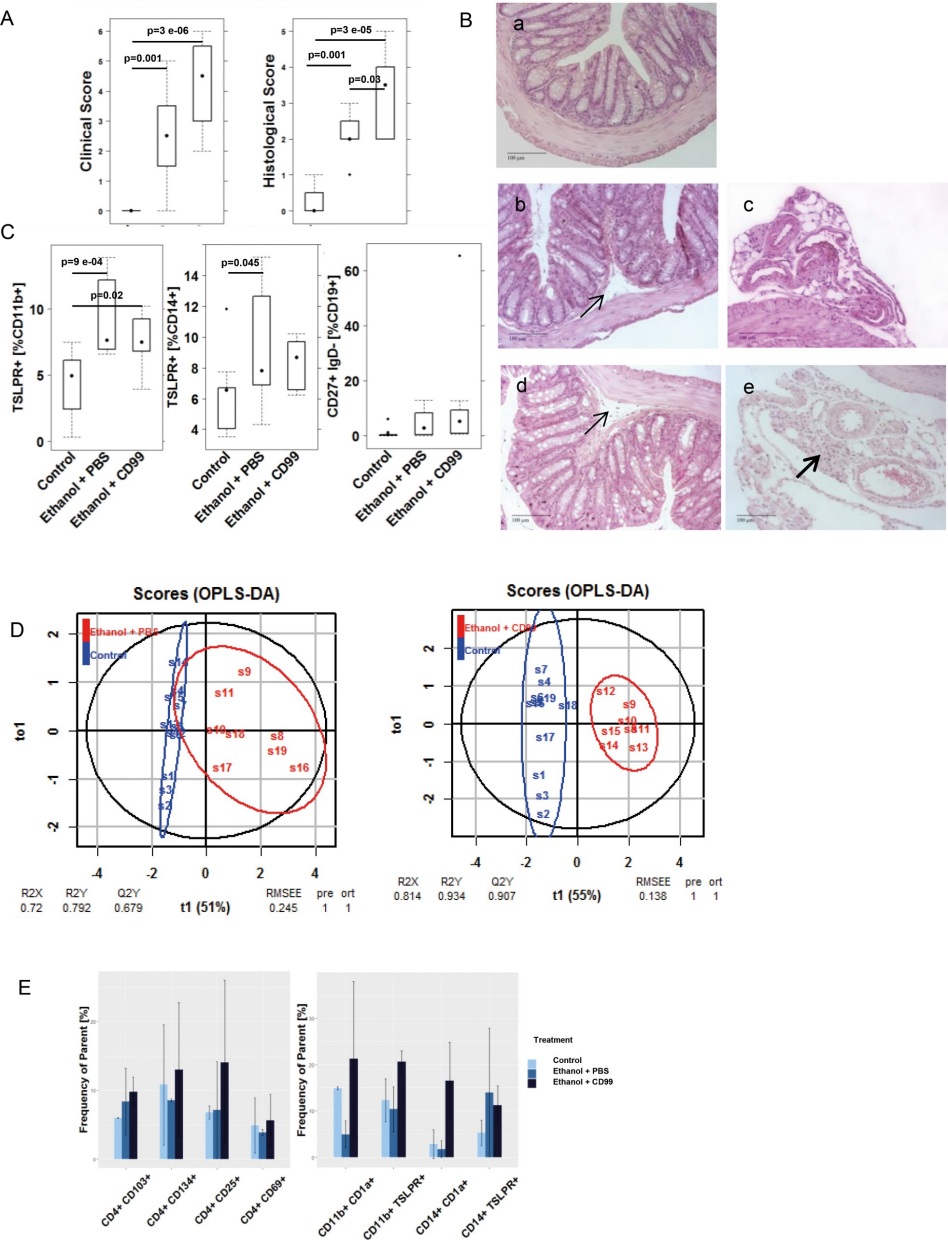
Additional challenge with CD99 aggravates the pathological phenotype of UC in NSG mice reconstituted with PBMC derived from patients with UC. (A) Clinical activity score and histological score of distal parts of the colon depicted as boxplot diagrams. NSG mice were reconstituted with PBMC from two different UC patients exhibiting autoantibodies against CD99. Mice were challenged with 10% ethanol on day 8, 30% ethanol on day 15 and 18 and additionally challenged with 10 μg of CD99 in PBS on day 8, 15 and 18. Sample sizes: Control n = 8; challenged control (Ethanol + PBS) n = 8; additionally challenged (ethanol + CD99) n = 8. (B) Photomicrographs of stained H&E paraffin sections from distal parts of the colon of NSG mice that had been treated as in A. (a) Unchallenged control, (b, c) challenged control (ethanol + PBS), (d, e) additionally challenged with CD99 (ethanol + CD99). Arrows indicate edema and influx of immune cells into the submucosa, bold arrows influx of inflammatory cells into the mesentery. (C) Frequency of human leucocytes isolated from spleen of NSG-UC mice and analyzed by flow cytometric analysis depicted as boxplot diagrams. (D) oPLS-DA analysis of control versus the ethanol + PBS and control versus ethanol + CD99 using the following parameters: Clinical score, colon score, histological score, frequencies of TSLPR expressing CD11b+ and CD14+ cells and switched B cells. (E) Frequencies of human leucocytes isolated from colons of mice that had been treated as described in A. Samples from each group were pooled for flow cytometric analysis. Mean values are given; error bars are s.d. Sample sizes: donors with anti-CD99 antibodies n = 2. Control n = 2; challenged control (Ethanol + PBS) control n = 2, additionally challenged (ethanol + CD99) n = 2.

To examine the role of autoantigens in an inflammatory environment *in vivo*, the effect of CD99 was analyzed in NSG-UC model [7, 9, 23]. NSG mice were reconstituted with PBMC from donors with UC and additionally challenged with CD99. Experiments were repeated twice with two different UC donors exhibiting elevated autoantibody levels (11.4 and 10·7 RFI). Mice were reconstituted with 4 x 10^6^ cells and eight days post reconstitution, the mice were divided into three groups: one was left unchallenged (unchallenged control) and the other two were pre-sensitized and challenged by rectal application of ethanol as described in Materiel and Methods. In previous experiments, a 50% concentration of ethanol was used to induce the symptoms of UC usually leads to inflammation. For concern that the effect of CD99 might be too subtle to be detected in a severe inflammatory background concentration of ethanol was reduced to 30% to induce a milder inflammation. One of the ethanol challenged groups (ethanol + CD99) was additionally challenged by intraperitoneal injection of CD99 (10μg in 200 μL PBS) on days 8, 15 and 18. The control challenged group (ethanol + PBS) received 200 μL PBS. Each group consisted of four animals. The onset of the disease was monitored by measuring body weight, and visual inspection of stools and the mice themselves. Symptoms were classified according to a clinical activity score. As expected the response to 30% ethanol was less pronounced compared to previous studies when animals had been challenged with 50% ethanol [7]. When 50% ethanol was used the mean value of the clinical activity score was 7.85 ±1.16 (mean±s.d.). When 30% ethanol was used the score reached a mean value of 2.5±1.6 (for complete data set see S4 Table). Mice additionally challenged with CD99 exhibited a significantly increased clinical activity score (CAS) as compared to the group solely challenged with ethanol (Fig 4A). The morphological changes of the colon were classified according to a histological score as described in Materials and Methods. As expected, the histological score in the control challenged group was not as high as previously observed when 50% ethanol was used. In these experiments, the histological score reached a mean value of 5.8±2.2 (mean±s.d.), whereas the histological score in mice challenged with 30% ethanol and reached a mean value of was 2.12±0·64 (Fig 4A). The additional challenge with CD99 resulted in a significantly increased histological score compared to the challenged control group. CD99 exposure resulted in a higher influx of immune cells into the mesentery and the development of slightly more pronounced edema (Fig 4B).

In order to examine the effect of additionally applied CD99 on human splenic leukocytes, cells were extracted from the spleen and subjected to flow cytometric analysis. As shown in Fig 4C, frequencies of TSLPR expressing CD11b+ and CD14b+ were significantly induced in ethanol challenged mice. Switched B cells (CD19+ CD27+ IgD-) were also increased; however the difference was not significant. In all other cellular populations no significant difference was observed between ethanol challenged mice and additionally CD99 challenged mice.

To analyze whether ethanol challenged mice and additionally CD99 challenged mice could be discriminated from the control group, an orthogonal partial least square discrimination analysis (oPLS-DA) was performed with the following parameters: Clinical score, colon score, histological score, frequencies of TSLPR expressing CD11b+ and CD14+ cells and switched B cells. As shown in Fig 4D the ethanol challenged group and the additionally CD99 challenged group were separated from the control group. Based on this analysis a difference could be observed between both groups. Evaluation of the model revealed that the additionally CD99 challenged group exhibited a higher R^2^X –value (0·814) and a lower RMSEE—value (0·138) when compared to the ethanol challenged group (0·72 and 0·245, respectively) indicating that the ability of this model to differentiate between the control group and the challenged groups is slightly higher in the group additionally challenged with CD99.

In order to examine the effect on human leucocytes in the colon these cells were isolated from the colon and subjected to flow cytometric analysis. As shown in Fig 4E, additional challenge with CD99 provoked an increase of activated CD4+ T-cells as indicated by an increase of frequencies of CD4+ CD134+ and CD4+ CD25+ T-cells. In addition, an increase of frequencies of mucosal T Cells (CD4+ CD103+) was observed. When subtypes of CD11b+ macrophages and CD14+ monocytes were analyzed, an increase of frequencies of CD11b+ CD1a+, CD11b+ TSLPR+, CD14+ TSLPR+ and CD14+ CD1a+. CD1a+ expressing monocytes and macrophages had previously identified as inflammatory markers in the human colon and in the mouse model [7, 22].

**Fig 5:**
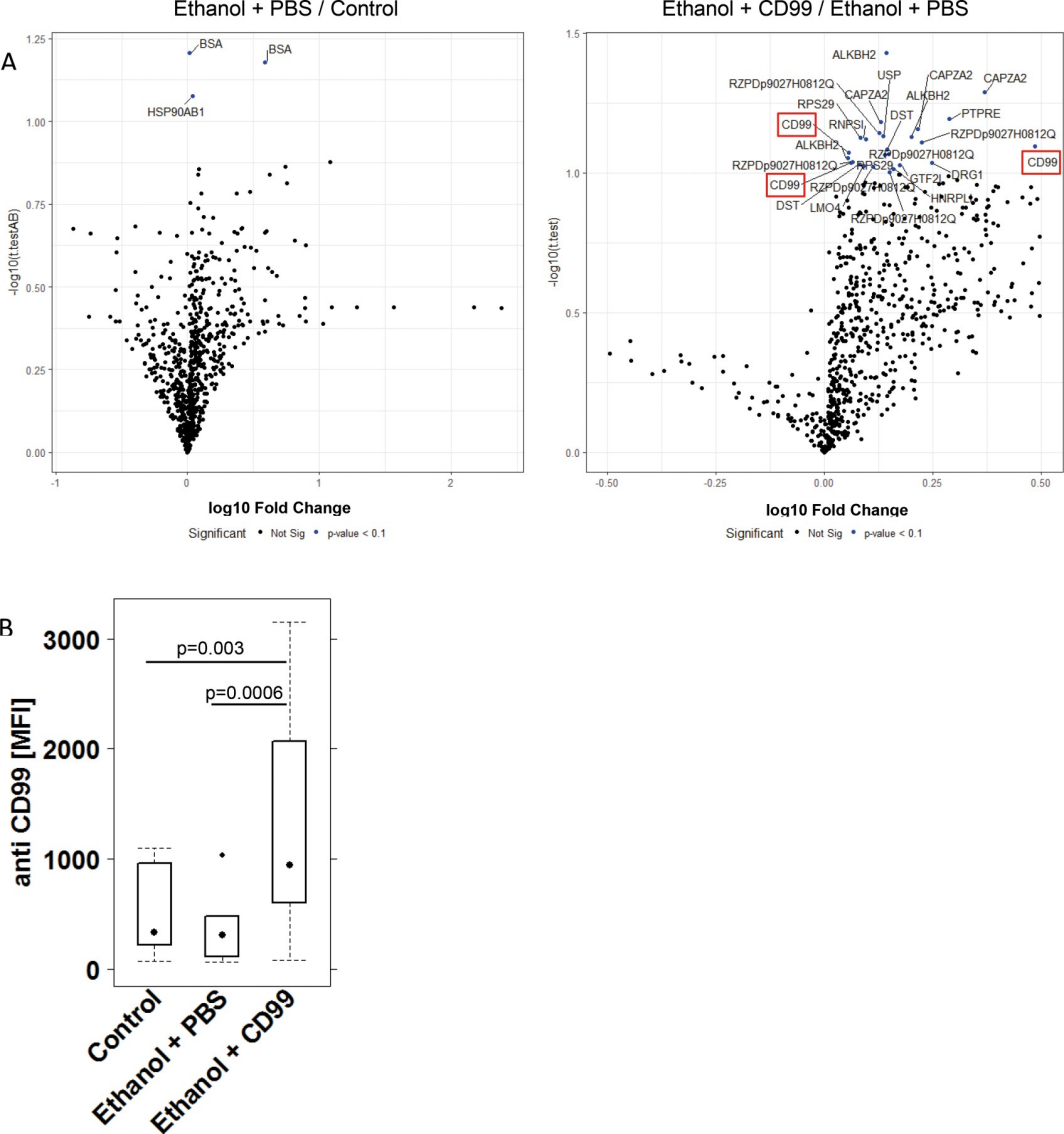
Additional challenge with CD99 leads to increased autoantibody levels. Mice were treated as described in Fig 4. Sera from mice were subjected to protein microarray analysis containing 192 antigens. (A) Changes in autoantibody levels depicted as volcano plots. A Student t- Test was performed to discriminate between the groups and changes with a trend to significance (p<0.1) are depicted in blue. (B) Changes in anti CD99 antibody expression levels depicted as a boxplot diagram. Sample size: Control n = 10, Ethanol + PBS n = 16, Ethanol + CD99 n = 13. Boxes represent upper and lower quartiles, whiskers represent variability and outliers are plotted as individual points. For comparison of groups, ANOVA followed by Tukey’s HSD was conducted.

To examine whether additional challenge with CD99 affected autoantibody levels sera from these mice were subjected to the same analysis as in previous experiments. This time the panel of antigens was reduced to those which had revealed significant elevated auto-antibodies (n = 192) in the previous study as described in Material and Methods. A student T-test was performed to determine significant differences; however, in light of the expected weak effect of CD99 on antibody levels the bar of significance was reduced to a p-value of < 0·1 to indicate changes with a trend to significance. Volcano plots revealed that challenge with ethanol as compared to control did not result in a significant increase of auto-antibodies with the exception of anti BSA and anti HSP90AB1 (Fig 5A). In sera from mice additionally challenged with CD99, however, autoantibody levels increased (Fig 5B). Elevated levels of antibodies against 28 proteins derived from 192 human cDNA expression clones were identified as described in Material and Methods six of which were detected repeatedly at different concentrations (ALKBH2 4x, CD99 3x, CAPZA2 3x, DST 2x, PTPRE 2x, RPS 2x and RZDp9027H0812Q 6x). Two different clones of CD99 were detected and when two different concentrations of these samples were combined for statistical analysis a significant increase of autoantibodies directed against CD99 was detected (Control (n = 10): 398 ± 323; mean MFI ± sd; Ethanol + PBS (n = 16): 358 ±316; Ethanol + CD99 (n = 13): 1363 ± 1336). Statistical analysis of all groups by ANOVA followed by TukeyHSD revealed a significant difference between the Control group versus Ethanol + CD99 p = 0·003 and Ethanol + PBS versus Ethanol + CD99: p = 0·0003).

### Auto antibody levels in the NSG-UC model

In order to examine the impact of a highly inflammatory environment on autoantibody levels NSG-UC mice were challenged with 50% ethanol. Two different patients in relapse were selected for reconstitution displaying a SCCAI score of five.

**Fig 6:**
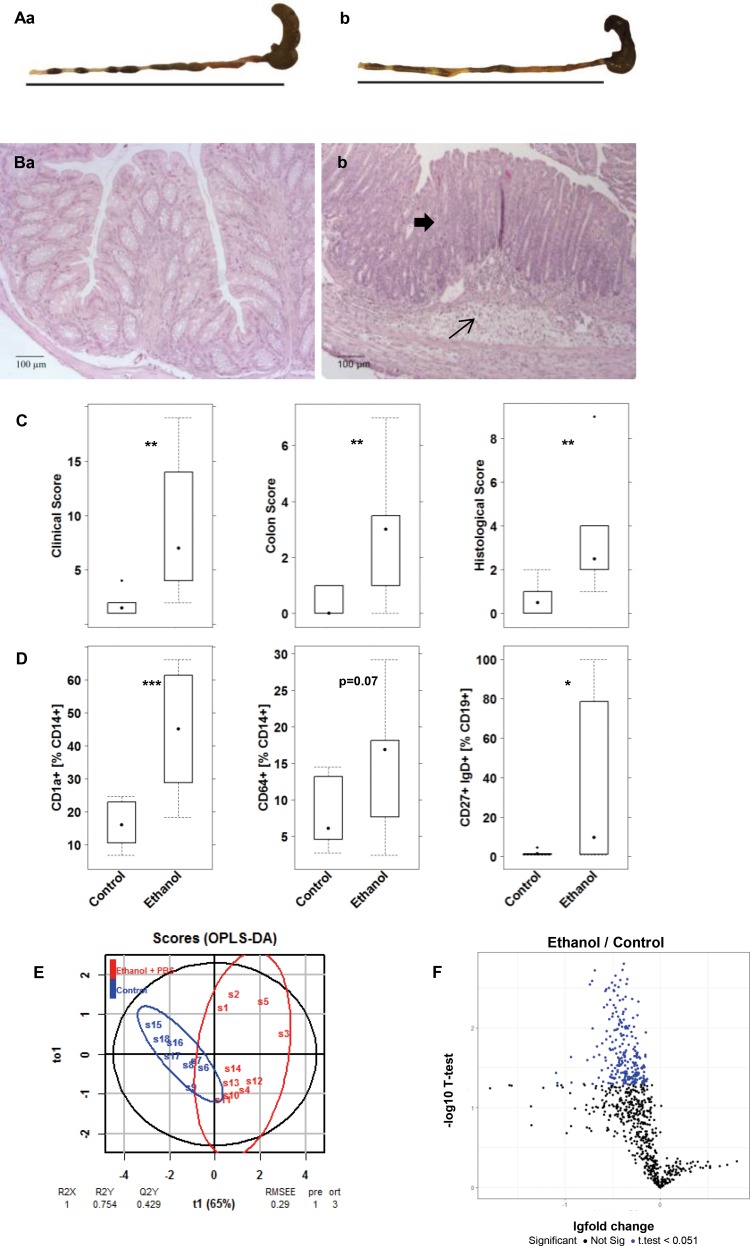
Decrease of autoantibody levels in highly inflammatory NSG mice. Mice were reconstituted with PBMC derived from two different UC patients, challenged with 10% ethanol at day 8, and 50% ethanol at days 15 and 18. (A) Macrophotographs of colons at autopsy. (B) Photomicrographs of H&E-stained sections of distal parts of the colon from mice. Arrows indicate edema and influx of inflammatory cells, bold arrows crypt loss. (C) Clinical-, colon- and histological scores of NSG-UC depicted as boxplot diagrams. (D) Frequency of human leucocytes isolated from spleen of NSG-UC mice and analyzed by flow cytometric analysis depicted as boxplot diagrams. Sample sizes: Control (n = 8); Ethanol + PBS (n = 10), Labels given on x-axes on the bottom row apply to all charts. Boxes represent upper and lower quartiles, whiskers represent variability and outliers are plotted as individual points. For comparison of groups, ANOVA followed by Tukey’s HSD was conducted. (E) oPLS discriminating analysis combining clinical-, colon, histological scores, frequencies of CD14+CD64+, CD11+ CD1a+, CD19+CD27+IgD+. (F) Expression levels of autoantibodies in sera of mice depicted as volcano plots. Mouse sera were subjected to protein microarray analysis using the targeted array presenting 192 proteins as described in Material and Methods. Student T-test was used to discriminate between groups, the threshold for significance was p = 0.05 and significant changes are depicted in blue.

Mice were reconstituted according to the previous experiment and separated into two different groups. The control group (Control n = 8) remained unchallenged whereas the other group was challenged with 50% ethanol (Ethanol n = 10) as described in material and methods. As observed previously mice developed pronounced symptoms and pathological phenotypes in response to ethanol (Fig 6, for complete data set see S5 Table). Macroscopic inspection of the colon indicated diarrhea (Fig 6Ab) in the ethanol challenged group and photomicrographs from distal parts of the colon revealed a mixed infiltrate of leukocytes into the mucosa and submucosa and edema, epithelial erosions and crypt loss in the ethanol challenged group (Fig 6Bb) as opposed to the control group which had a healthy phenotype (Fig 6Ba). The clinical-, colon and histological scores increased significantly (Fig 6C). Analysis of human leukocytes isolated from the spleen revealed an increase in frequencies of CD14+ CD1a+ and CD14+ CD64+ both of which had been previously identified as markers of inflammation [8]. In addition immature B cells (CD19+ CD27+ IgD+) were found to be increased upon challenge with ethanol (Fig 6D). The most pronounced inflammatory markers (clinical-, colon- and histological score and frequencies of CD14+ CD1a+ monocytes) were combined and analyzed in an oPLS-DA analysis. As shown in Fig 6E this model had an optimal coefficient of determination value (RX^2^) of 1 and a low root mean square error of estimation (RMSEE) value of 0·29 further corroborating the pronounce inflammation in the ethanol challenged groups.

Like in the previous experiment sera from these mice were subjected to autoantibody level analysis (Control n = 6; Ethanol n = 10). Serum levels of autoantibodies in the NSG-UC mouse model as indicated in the volcano plot (Fig 6F). Levels of CD99 autoantibodies were significantly reduced (p value < 0·05).

## Discussion

Most studies aiming to determine autoantibodies in UC rely on selected antigens that have previously been found to be elevated in other auto immune diseases such as in granulomatosis-with-polyangitis for which cANCA is an established biomarker. As our previous study suggested that autoimmunity might play a role in UC we conducted a broad screening of autoantibodies using high density protein-microarrays. Rather unexpectedly, the screening of UC patients versus non-UC controls yielded a remarkable 697 significantly different reactive antigens. Autoantibodies directed against CD99 emerged as a biomarker to discriminate between non-UC subjects and UC patients. The fact that autoantibody levels were also significantly increased in donors prior to diagnosis and differed in samples closer to diagnosis as compared to samples with a longer time interval to diagnosis suggests leakiness in peripheral and/or central tolerance. Although it is not possible to trace back whether the donors already experienced light symptoms of UC, this finding indicated that autoantibodies per se might not be pathological. The same phenomenon has been observed in rheumatoid arthritis where asymptomatic donors exhibited autoantibodies against cyclic citrullinated peptide etc. years before the onset of disease symptoms [24]. Given that the cDNA library did not cover the entire genome presumably expressed in humans, this study cannot claim that all autoantigens were discovered and important autoantigens still might missing. Although this study is limited with regard to this aspect it constitutes an improvement compared to previous studies as it suggests a general break in tolerance which has not been observed previously. The presence of autoantibodies, however, indicates a breach in the central and/or peripheral tolerance and the crucial questions are: What causes the leakiness in tolerance and could these autoantibodies contribute to the pathogenesis in UC? One route to enhanced exposure to autoantigens is autophagy. Autophagy refers to a mechanism by which immune cells generate energy in situations of stress, starvation, hypoxia, protein aggregates, or bacterial and viral infections through degradation and recycling of cellular components. During this process, intracellular antigens can be delivered to MHC class II; however, whether the presentation results in an immunological silent response as in the case of tolerance or induces the generation of inflammatory autoantibodies is dependent on the inflammatory milieu.

Three genes have been identified at UC susceptibility loci which might be involved in antibody generation and development of pathological auto-antibodies: IL10, IL23R and FCGR2A/C [25].

IL-10 is an important anti-inflammatory cytokine and IL-10R polymorphisms are associated with very early-onset ulcerative colitis [26]. The IL-23 TH17 axis; on the other hand regulates autoantibody activity by suppressing sialylation and thereby determines the onset of autoimmune disease e.g. rheumatoid arthritis. As autoantibodies precede the onset of disease the IL-23 TH17 axis might unlock a preexisting lack of tolerance [27]. It is noteworthy that we find the signature cytokine of Th17 cells, IL- 17a, as a significantly elevated cytokine in serum of UC patients. Finally, FcγRII A and C belong to the activating type of IgG receptors which upon engagement induce pleiotropic biological responses to include the secretion of pro-inflammatory cytokines such as IL-6 and IL-8 [28–30]. In addition, activation of FcγR1 on monocytes triggers differentiation into immature dendritic cells that induce autoreactive T cell responses [31]. We have found in previous studies that CD14+ CD64 (FcγR1) + monocytes are elevated in colon of UC patients. In addition, immune profiling of UC patients identified a subgroup of patients characterized by elevated levels of CD14+ CD64+ monocytes [32].

*In vitro*, CD99 protein added into cell culture did not activate CD4+ T cells and caused the release of IL-10 indicating the induction of tolerance. This is in agreement with studies which characterized autoantigens as important modulators of inflammation. Peptides of the heat shock proteins HSP 60 and 70 e.g. have been shown to induce regulatory T-cells, and to suppress type 1 diabetes or autoimmune arthritis in mice [33, 34]. Following this rationale, the HSP60 derived peptide DiaPep277 has been developed into a therapeutic for type 1 diabetes with proven efficacy in a phase II clinical trial [35]. Thus, the presence of antigens might not necessarily lead to activation of T cells, however, the presence of antibodies to these antigens and the activation of FcγRIIA/C or FcγRI might overcome the tolerogenic response.

*In vivo*, autoantibodies were also detected in the NSG-UC animal model. The observation that they also were found in the unchallenged control which exhibited no symptoms of inflammation suggests, that the presence of autoantibodies is not sufficient to induce pathological changes in this model. In a mild inflammatory background, additional challenge with CD99, however, led to increased clinical activity- and histological scores and antibodies directed against CD99. Thus, autoimmunity might contribute to inflammation in this model. This assumption is also corroborated by the observation that frequencies of TSLPR expressing monocytes and macrophages were elevated. TSLPR expressing antigen presenting cells induce Th2 characterized inflammation, M2 differentiation and fibrosis upon exposure to TLSP thereby shifting the pro-inflammatory response to immune modulating and wound healing processes [36–39]. Previous results in our animal model had shown that TSLP levels were slightly elevated in response to challenge with ethanol (data not shown); however, TSLP cannot exert its activity in this model as it has been found to be species specific [40].

In a highly inflammatory background, levels of autoantibodies were significantly decreased in sera of challenged mice.

The decrease observed in the mouse model was accompanied by an increase of CD14+ CD64+ (FcγR1) monocytes suggesting that the antibodies might be engaged and internalized during the inflammatory processes in the NSG-UC mouse model. Due to a limited amount of IgG which cannot be replenished during the days of active inflammation, the concentration of IgG decreases.

## Conclusion

We have shown that autoantibodies are significant markers of an ongoing inflammation in UC, it has, however, to be further elucidated whether and to what degree these autoantibodies promote inflammation as they may merely be an indication for the inability to control inflammation. Results from the NSG-UC model suggest that autoantibodies might promote inflammation and might partially explain the requirement of diseased donors in this model.

## Supporting information

S1 Fig Gating strategy. (DOCX)

S1 Table List of autoantibodies. (XLSX)

S2 Table List of autoantibodies longitudinal study. (XLSX)

S3 Table Complete data set in vitro experiment. (XLSX)

S4 Table Complete data set animal study 1. (XLSX)

S5 Table Complete data set animal study 2. (XLSX)

S6 Table Antibodies used for flow cytometric analysis. (DOCX)

S7 Table Cellular markers used for flow cytometric analysis. (DOCX)
