# Supplementary material for: Autoantibodies as diagnostic markers and potential drivers of inflammation in ulcerative colitis
Source: PLoS One. 2020 Feb 12;15(2):e0228615. doi: 10.1371/journal.pone.0228615 (PMC7015398; doi:10.1371/journal.pone.0228615)
Supplement: S1 Fig — (DOCX) [file pone.0228615.s001.docx]

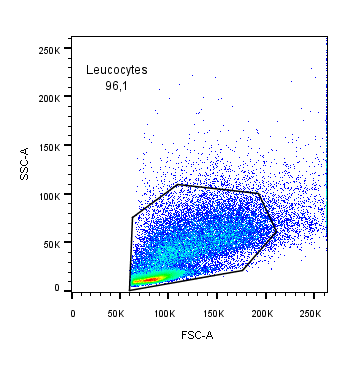

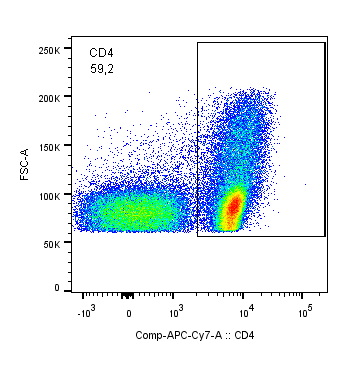

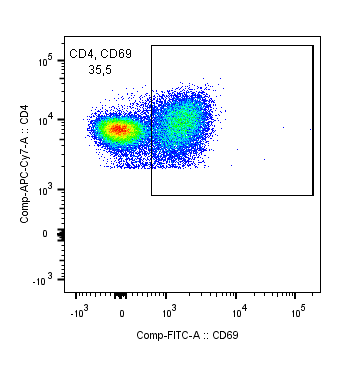

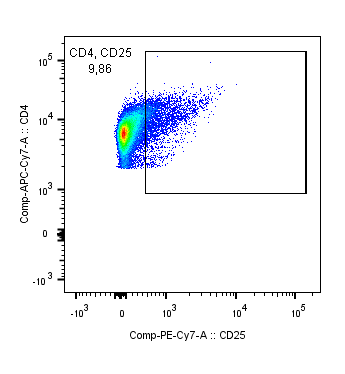

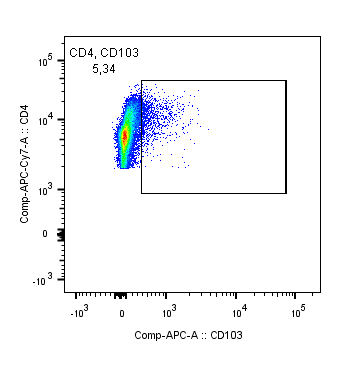

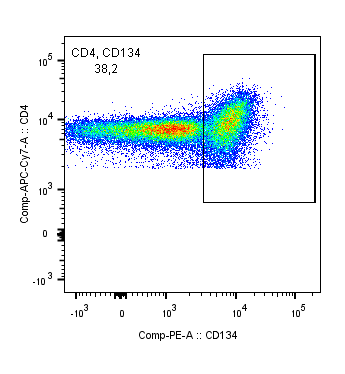


CD4+ CD25+

CD4+

CD4+ CD134+

CD4+ CD103+

CD4+ CD69+

CD25+ CD127^low^ CD25+


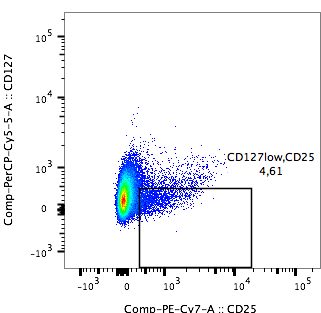


A

Leukocytes

B


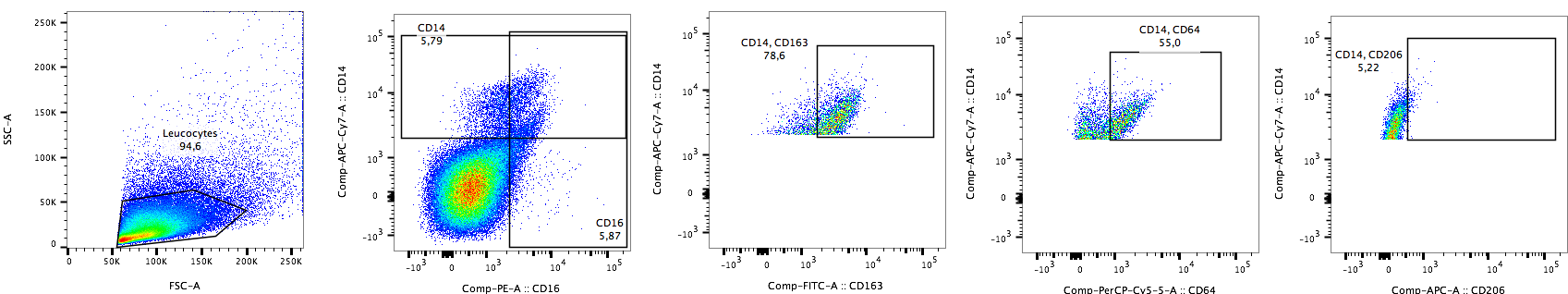


CD14+ CD1a+

CD14+ CD64+

CD14+TSLPR+

CD14+


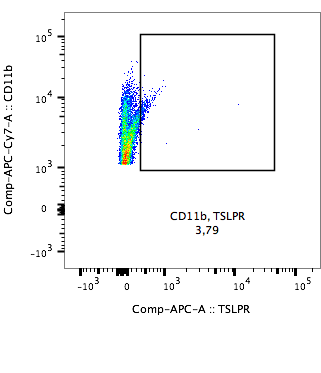

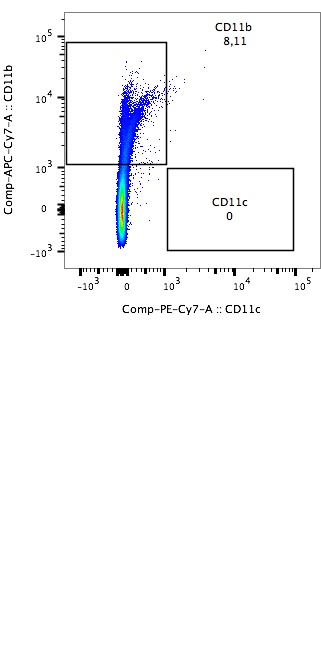


CD11b+

CD11b+ TSLPR+

CD19+

CD19+ CD27±

CD19+ CD27- IgD±

CD19+ CD27+ IgD±

CD19+ CD38+

C

CD4+ CD69+

CD4+ CD134+

CD4+ CD103+

CD4+ CD25+

CD4+

CD11b+

CD11b+ CD1a+

CD11b+ TSLPR+

CD14+

CD14+ CD1a+

CD14+TSLPR+

**Figure S1 Gating strategy** (A) Human PBMC Activated T cells. (B) Human leukocytes isolated from mouse spleen. (C) Human leukocytes isolated from mouse colon. Cells were labeled as described in Material and Methods using antibodies listed in Supplementary Material Table S6.
