# Supplementary material for: Autoantibodies as diagnostic markers and potential drivers of inflammation in ulcerative colitis
Source: PLoS One. 2020 Feb 12;15(2):e0228615. doi: 10.1371/journal.pone.0228615 (PMC7015398; doi:10.1371/journal.pone.0228615)
Supplement: S6 Table — (DOCX) [file pone.0228615.s007.docx]

**Table S6 Antibodies used to label human leukocytes**

| **Surfacemarker** | **Colour** | **Clone** | **Cat # RRID #** |
| --- | --- | --- | --- |
| CD19 | Peridine-chlorophyll-protein complex cyanine dye (PerCP-Cy^TM^ 5.5) | HIB19 | **BioLegend Cat# 302230, RRID:AB_2073119** |
| CD38 | Phycoerythrin (PE) | HB-7 | **BioLegend Cat# 356603, RRID:AB_2561899** |
| CD27 | Pe-Cy7 | LG.3A10 | **BioLegend Cat# 356411, RRID:AB_2562257** |
| IgD | Fluorescin isothiocyanate (FITC) | LA6-2 | **BioLegend Cat# 348217, RRID:AB_11204072** |
| CD4 | Allophycocyanin (APC)- Cy7 | OKT4 | **BioLegend Cat# 317417, RRID:AB_571946** |
| CD8 | PerCP-Cy^TM^ 5.5 | HIT8a |  |
| CD103 | APC | Ber-ACT8 | **BioLegend Cat# 350215, RRID:AB_2563906** |
| CD14 | APC-Cy7 | HCD14 | **BioLegend Cat# 325619, RRID:AB_830692** |
| TSLPR | APC | 1B4 | **BioLegend Cat# 322807, RRID:AB_2085327** |
| CD1a (biotin)/secondary Ab streptavidin | FITC | HI149 | **BioLegend Cat# 300112, RRID:AB_389344** |
| CD64 | PerCP-Cy^TM^ 5.5 | 10.1 | **BioLegend Cat# 305023, RRID:AB_2561585** |
| CD163 | FITC | GHI/61 | **BioLegend Cat# 333617, RRID:AB_2563093** |
| CD206 | APC | 15-2 | **BioLegend Cat# 321109, RRID:AB_571884** |
| CD16 | PE | 3G8 | **BioLegend Cat# 302007, RRID:AB_314207** |
| CD11b | APC-Cy7 | ICRF44 | **BioLegend Cat# 301341, RRID:AB_2563371** |
| CD11c | PE-Cy7 | 3.9 | **BioLegend Cat# 301607, RRID:AB_389350** |
| CD69 | FITC | FN50 | **BioLegend Cat# 310903, RRID:AB_314838** |
| CD25 | PE/Cy7 | BC96 | **BioLegend Cat# 302611, RRID:AB_314281** |
| CD134 (Ox40) | PE | Ber-ACT35 | **BioLegend Cat# 350003, RRID:AB_10641708** |
| CD127 | Per CP Cy5 | A019D5 | **BioLegend Cat# 351321, RRID:AB_10900253** |
